# Can we use normal saline stored under stress conditions? A simulated prehospital emergency medical setting

**DOI:** 10.1016/j.heliyon.2023.e20377

**Published:** 2023-09-25

**Authors:** Ousama Rachid, Mohammed Akkbik, Alaaldin M. Alkilany, Ahmed Makhlouf, Loua Al Shaikh, Guillaume Alinier

**Affiliations:** a College of Pharmacy, QU Health, Qatar University, Doha, Qatar; b Central Laboratories Unit, Office of VP for Research & Graduate Studies, Qatar University, Doha, Qatar; c Hamad Medical Corporation Ambulance Service, Doha, Qatar; d School of Health and Social Work, University of Hertfordshire, Hatfield, UK; e Weill Cornell Medicine-Qatar, Doha, Qatar; f Faculty of Health and Life Sciences, Northumbria University, Newcastle upon Tyne, UK

**Keywords:** 0.9% sodium chloride, Normal saline, Stability, Temperature, Prehospital, Emergency medical setting

## Abstract

**Background:**

Data on stability and suitability to use normal saline stored under stress conditions in ambulances is lacking.

**Objective:**

We aimed to study the impact of exposure to extreme temperature variations on normal saline stability and compatibility with its packaging.

**Methods:**

Normal saline in 96 polyolefin bags were exposed to continuous temperature of 22, 50, and 70 °C or to a cyclic temperature of 70 °C per 8 h and 22 °C per 16 h. The bags were sampled at 12, 24, 48 and 72 h and at 1, 2, 3, and 4 weeks in the short- and long-term experiments, respectively. Solution inside the bags was evaluated for any evidence of crystallization, discoloration, turbidity, or pH changes. A sample of normal saline was withdrawn from each bag to analyze sodium and chloride levels.

**Results:**

Precipitation, discoloration, or turbidity were not observed in the solution inside normal saline bags. The average pH was 5.59 at 22 °C, 5.73 at 50 °C, 5.86 at 70 °C and 5.79 at cyclic exposure. In the short- and long-term experiments, sodium and chloride concentrations were within 100.2–111.27% and 99.04–110.95%, respectively. Leaching of the plastic components in the polyolefin bag into the normal saline solution was not detected.

**Conclusions:**

Sodium and chloride levels of normal saline were stable and compatible with polyolefin bags stored in simulated continuous and cyclic extreme temperatures for around one month. The effect of storage in the cabinet of operational ambulance vehicles during different seasons in arid countries is yet to be evaluated in real-world conditions, to further confirm our results.

## 1 Introduction

In the USA, 0.9% sodium chloride intravenous (IV) fluid (normal saline) is widely used with more than 200 million liters administered annually [1,2]. Since its inception, normal saline has saved lives and remains the most widespread crystalloid commonly used in patient care [3]. It is administered for hydration purposes [4,5]. The utilization of cold intravenous normal saline for the induction of therapeutic hypothermia has been reported [6,7]; while warmed normal saline can be infused to patients with hypothermia [8,9]. Indications of normal saline infusions also include adding it as a pharmaceutical vehicle and diluent for the infusion of compatible drug additives [10].

Normal saline solution contains sodium and chloride in equal concentrations. Within each 1000 mL of United States Pharmacopeia (USP) 0.9% sodium chloride, there is 154 mEq of sodium ions and 154 mEq of chloride ions. Its osmolarity is 308 mOsmol/L and it has a pH of 5.6 (range 4.5–7.0) [11]. Sodium ion is the main electrolyte of extracellular fluid, playing a major role in the distribution of fluids and other electrolytes. Chloride ion is employed as a buffering agent within lungs and tissues, where it aids in facilitating binding of hemoglobin to oxygen or carbon dioxide [12].

To ensure safety, efficacy, and stability of medications, the USP provides guidelines for the appropriate storage and distribution conditions of each medication in its designated monograph. However, in a prehospital emergency medical service (EMS) setting, following such guidelines is sometimes a challenging task due to the nature of the work and the surrounding environment over which crews have little or no control. Medications in EMS vehicles are exposed to uncontrolled environmental factors such as light, vibration, humidity, and temperature variations, the latter being the most studied factor. We have evaluated the stability of the EMS medication, epinephrine, stored in excessively high temperatures, which lead to significant degradation and therefore potential under-dosing in life-threatening situations [13]. Other studies were also conducted to evaluate the stability of epinephrine in different dosage forms beyond recommended storage conditions [[14], [15], [16]]. Amiodarone, rocuronium, fentanyl, succinylcholine, and epinephrine were found to be stable when stored over one year onboard an EMS vehicle under real-life ambient temperature which ranged from 13.9 °C to 33.9 °C [17]. Other medications such as ketamine, diazepam, and midazolam were relatively stable although subjected to beyond recommended conditions for a long-term exposure of up to three months for diazepam and midazolam and six months for ketamine of EMS deployment in high-heat environment with a recorded mean kinetic temperature of up to 27.1 °C for ketamine and 31.6 °C for diazepam and midazolam [18,19]. Stability of medications stored in air rescue vehicles such as helicopters was evaluated and found to be affected (although remained above the 90% acceptable limit) due to exposure to temperatures outside manufacturers' storage recommendations with recorded temperatures of up to 38.1 °C [20]. A literature review of the prehospital medications concluded that the temperatures recorded does not follow the USP guidelines and further studies are needed to assess the impact of storage conditions and uncontrolled environmental factors of an EMS on medication stability [21]. In the Gulf Cooperation Council region including Qatar, the rise in temperature and relative humidity may reach >50 °C and 95%, respectively, as reported by the Qatar Civil Aviation Authority. Although the national Ambulance Service benefits from a very modern fleet of vehicles carefully designed and equipped, all with air conditioning, heat and humidity quickly fill the vehicle as soon as the vehicle's doors are opened, or if the engine is turned off, and as such anything stored inside the vehicle is exposed to environmental variations [22]. Data on the thermal stability of normal saline, in prehospital settings in the Middle East and North Africa (MENA) region, are lacking [23].

Therefore, there is a need to assess the effect of specific environmental conditions in the prehospital environment such as temperature and duration of exposure on stored normal saline solution in an ambulance. In addition, the effect of thermal stability on the type of plastic in the packaging of normal saline solution should be evaluated for compatibility and safety [24]. This packaging may include polyvinyl chloride or polyolefin (PO), produced from olefin or alkene polymers, which is commonly used in IV infusion containers in the US and worldwide [25]. The aim of this study was to assess the effect of exposure to short- and long-term temperature variations on the stability of normal saline products. This is the first report to determine the effect of varied thermal exposure in simulated prehospital settings on normal saline solution, and to evaluate the compatibility between polyolefin (PLÜMAT) fluid bags with normal saline, as defined as a solution free from plastic components.

## 2 Material and methods

### 2.1 Apparatus and conditions

Sodium and chloride levels were determined by an ion exchange chromatography (IEC) system using a Metrohm 850 Professional IEC (Herisau, Switzerland) [[26], [27], [28]]. The conditions of the IEC system are summarized in Table 1.

**Table 1:** Optimized parameters for ion exchange chromatography measurement.

Parameter	Value
Eluent	3.2 mM Na_2_CO_3_/1.0 mM NaHCO_3_ (anions) 0.7 mM dipicolinic acid (DPA)/2.8 mM HNO_3_ (cations)
Flow	0.7 mL/min (anions) 0.9 mL/min (cations)
Columns	Metrosep A Supp 5–150/4.0 (anions) Metrosep C_4_ – 150/4.0 (cations)
Temperature	30 °C
Sample loop	250 μL
Injection volume	20 μL (anions) and 10 μL (cations)
Recording Time	22.0 min (anions) and 13.0 min (cations)
Suppressor	0.1 M H_3_PO_4_
Detector	Conductivity detector

### 2.2 Reagents and materials

Stock standard solutions of sodium and chloride (1000 mg/L) for calibration curves were purchased from Sigma-Aldrich (Darmstadt, Germany). Analytical grade of dipicolinic acid, sodium bicarbonate, sodium carbonate, nitric acid, and phosphoric acid were obtained from Sigma-Aldrich (Darmstadt, Germany). All the eluents were filtered by Handling Systems (Kontes, Vineland, NJ) under vacuum using 0.45 μm nylon solvent filter (Pall, Michigan, USA), and degassed using XUB digital ultrasonic bath (Royston, UK) prior to their use. Ultrapure water was used to prepare all the solutions using a Milli-Q water purification system (Millipore, Biller ica, MA, USA).

### 2.3 Sample collection and preparation

**Fig. 1:**
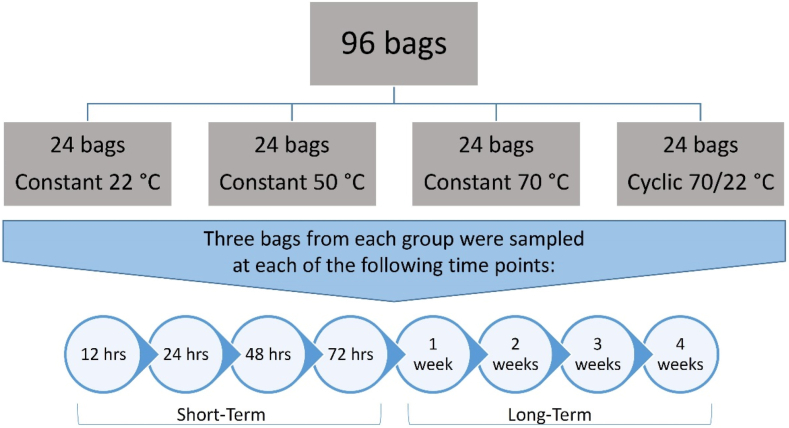
Diagram of the normal saline groupings and sampling time points.

In this study, 0.9% sodium chloride in 500 mL polyolefin (PLÜMAT) fluid bags were obtained commercially from Qatar Pharma (Doha, Qatar) with the same batch number (BN:1929013008). Ninety six fluid bags were classified into four groups of 24 bags each (Fig. 1) on the basis of temperature exposure: for short-term (12, 24, 48, and 72 h) and long-term (1, 2, 3 and 4 weeks) storage at either continuous temperature of 22, 50, and 70 °C or cyclic temperature of 70 °C for 8 h and 22 °C for 16 h. Using a needle and a 1 mL sized-syringe, samples of normal saline solution were withdrawn from the access port of each fluid bag and stored at 4 °C until analysis. The analysis of sodium and chloride ions was achieved by diluting a 20 μL of the withdrawn out-of-fridge sample in 12 mL distilled water and injected into the ion exchange chromatography instrument (Metrohm, 850 Professional IEC).

### 2.4 Physical stability

The physical stability of normal saline samples was examined by visual inspection. Prior to each sampling time, the solution was allowed to equilibrate to room temperature, mixed by inversion, and visually inspected. The samples were evaluated against a black and white background for any visible signs of particulate matter, cloudiness, or color change. In addition, the pH change of samples was measured by Oakton pH 2700 pH/mV/°C/°F Meter (Oakton Instruments, Illinois, USA) at room temperature. Standard solutions of pH 4.00, 7.00, and 10.00 were used for calibration of the pH meter.

### 2.5 Migration of plastic components

The potential adsorption profile of plastic components (olefinic monomers, plasticizers, polymerization initiators, and stabilizing additives) migrated from PO surface to normal saline solution was monitored by UV–Vis spectral scanning (200–500 nm) using a Varian Cary 50 Conc UV–Vis Spectrophotometer (Varian, Victoria, Australia).

## 3 Results

**Fig. 2:**
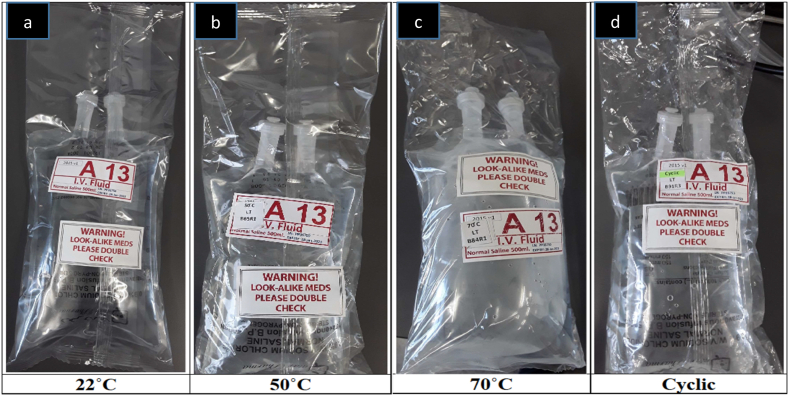
Bulging effect of constant temperature at a) 22, b) 50, C) 70 °C and d) cyclic temperature on the polyolefin (PLÜMAT) packaging bags of normal saline.

Among different normal saline sample groups subjected to different temperature and exposure time combinations, physical characteristics such as solution clarity, lack of visible precipitations, and lack of color changes were observed, suggesting that all the samples maintained their physical stability. The PO (PLÜMAT) bags bulged slightly at 50 °C and largely at 70 °C and cyclic temperature exposure (Fig. 2) which was attributed to vaporized water from the prolonged heating (over 28 days).

**Fig. 3:**
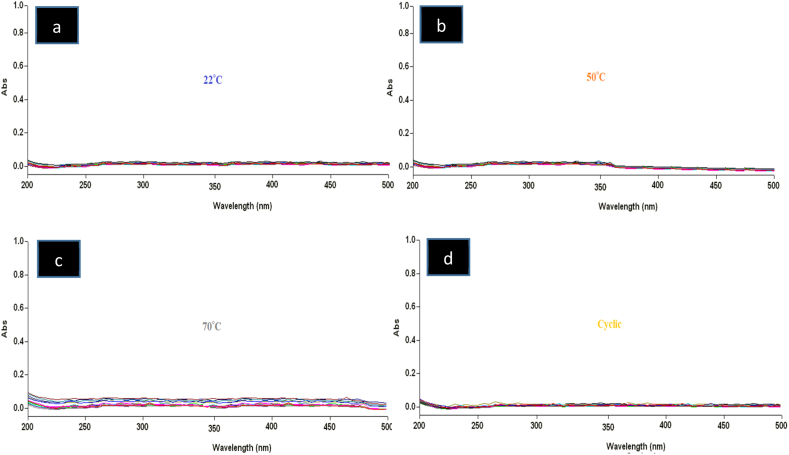
UV–Vis absorbance of normal saline solution in 500 mL polyolefin (PLÜMAT) bags stored at different storage conditions a) 22, b) 50, c) 70 °C, and d) cyclic temperature variation over 28 days.

Analyses of leached plastic components (olefinic monomers, plasticizers, polymerization initiators, and stabilizing additives) were performed by scanning a UV–Vis absorption spectroscopy of normal saline solution from 200 to 500 nm based on Chang et al. (2010) method with minor modifications (Fig. 3) [25]. Normal saline solution in polyolefin (PLÜMAT) fluid bags incubated at 50 °C, 70 °C and cyclic temperature variation did not have any absorbance over the 200–500 nm range as compared to normal saline solution that was stored at 22 °C. None of the plasticizers and other additives that are known to leach from plastic components were found.

**Fig. 4:**
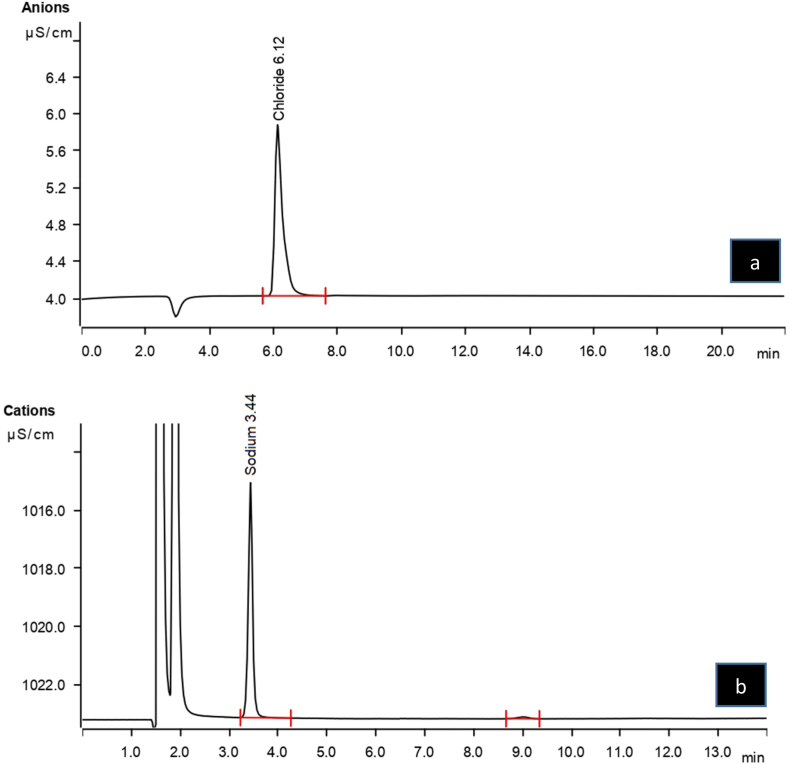
Typical chromatograms of a) anion exchange chromatography for chloride at 10 mg/L by eluent with 3.2 mM sodium carbonate and 1.0 mM sodium bicarbonate and b) cation exchange chromatography for sodium at 10 mg/L by eluent with 0.7 mM dipicolinic acid and 2.8 mM nitric acid.

Thermal stability of normal saline solution in polyolefin (PLÜMAT) container at different temperatures was assessed by examining the percentage change from the initial concentrations of chloride (Fig. 4a) and sodium (Fig. 4b) (defined as 100%), and subsequent sample concentrations were expressed as the percentage of the initial concentration (mean ± SD) of three replicate samples. Normal saline solution had an initial pH of 4.5–7.0 and its thermal stability in short-term and long-term studies is summarized in Table 2, Table 3, respectively.

**Table 2:** Remaining percentage of sodium and chloride (n = 48) in short-term stability study.

T (°C)	Na⁺ levels (%Remaining±SD)				C $l^{-}$ levels (%Remaining±SD)			
	Time (Hours)				Time (Hours)			
	12	24	48	72	12	24	48	72
22	100.9 ± 0.8	100.2 ± 0.9	100.6 ± 0.5	100.8 ± 0.7	99.3 ± 0.7	99.0 ± 0.6	99.5 ± 0.7	99.4 ± 0.4
50	104.3 ± 1.5	104.7 ± 1.9	104.7 ± 0.8	105.3 ± 0.3	101.3 ± 1.8	100.4 ± 1.7	100.1 ± 0.7	101.8 ± 0.7
70	106.3 ± 0.8	110.2 ± 0.8	110.1 ± 1.5	110.7 ± 0.8	100.1 ± 1.4	101.7 ± 1.3	102.1 ± 1.7	102.0 ± 0.7
Cyclic	107.1 ± 1.6	105.7 ± 1.1	105.8 ± 1.6	107.9 ± 0.7	100.5 ± 1.6	99.8 ± 1.8	100.0 ± 1.6	100.9 ± 0.9

**Table 3:** Remaining percentage of sodium and chloride (n = 48) in long-term stability study.

T (°C)	Na⁺ levels (%Remaining±SD)				C $l^{-}$ levels (%Remaining±SD)			
	Time (Weeks)				Time (Weeks)			
	1	2	3	4	1	2	3	4
22	103.4 ± 0.6	103.7 ± 2.3	103.0 ± 1.7	101.9 ± 0.9	101.9 ± 1.1	101.6 ± 1.7	99.0 ± 0.9	100.0 ± 0.8
50	102.4 ± 0.8	101.9 ± 0.8	109.8 ± 1.8	104.5 ± 4.2	100.8 ± 1.0	101.6 ± 1.3	109.9 ± 1.3	102.8 ± 2.0
70	105.8 ± 1.1	104.7 ± 2.5	109.9 ± 2.0	111.3 ± 2.6	103.7 ± 0.8	104.0 ± 2.4	108.8 ± 2.4	111.0 ± 1.6
Cyclic	103.7 ± 1.8	104.5 ± 1.6	105.4 ± 1.5	105.6 ± 1.7	103.7 ± 1.4	103.3 ± 1.1	105.5 ± 0.8	105.0 ± 1.6

## 4 Discussion

The stability of EMS medications including intravenous fluids stored under stress conditions is a major concern that has been evaluated in many studies. Helm M et al. evaluated the temperature effect on EMS medications in real rescue missions involving helicopter, ambulance and emergency physician transport vehicles [29]. They logged temperatures over two months in the summer and two months in the winter seasons in south Germany. The temperatures reported varied from −13.2 °C to +50.6 °C and all the rescue vehicles were exposed almost half of the time to temperatures exceeding the recommended maximum storage temperature of 25 °C. The authors’ recommendations included temperature controlled-storage boxes; however, they did not analyze the medication content changes. Specific medications used in a National Park Service showed different degrees of degradation at extremes of temperature in a heat-dependent or –independent manner [30].

In addition to thermal stability, compatibility is a major factor to be considered in the intravenous fluids used in the prehospital setting of a country with an arid climate, which can be very hot and humid. This study examined both thermal stability and compatibility for up to 28 days in different temperature storage conditions; however, we could not at the same time simulate high levels of environmental humidity. To our knowledge, there is limited or no published data specifically for the explicit effect of extreme thermal storage conditions for normal saline solution in polyolefin (PLÜMAT) bags. The compatibility between polyolefin (PLÜMAT) container with normal saline solution that has the potential of leaching plasticizer or other polymer matrix components from polyolefin (PLÜMAT) bags into the fluid was studied. The bags were incubated for 28 days at 22, 50, 70 °C and cyclic temperature variation. The UV–Vis spectral absorption of the normal saline solution from collected samples did not exhibit absorbance at 226 nm indicating the leaching of additives such as derivatives of phthalic acid [31] or at 320 nm peak for other plastic components [25]. Chang et al. made explicit discussion on normal saline with anticancer agent in PVC and polyolefin infusion bags [25]. Our findings suggest the absence of any leaching of plastic components or additives (phthalic acid) from polyolefin (PLÜMAT) bags in normal saline solution (Fig. 3). The polyolefin (PLÜMAT) container was found to be compatible with normal saline intravenous solution. This result is in agreement with Trissel et al. who had studied compatibility of seven drugs with polyolefin infusion bags [32].

Results revealed that the normal saline solution in 500 mL polyolefin (PLÜMAT) bags were physically stable (no visible particulate, no cloudiness, and stable pH) throughout this study. The mean pH values were 5.59 ± 0.08 at 22 °C, 5.73 ± 0.04 at 50 °C, 5.86 ± 0.02 at 70 °C, and 5.79 ± 0.03 at cyclic temperature exposure, all of which fall within the acceptable pH value range of 4.5–7.0. This thermal stability measure is important in assessing the remaining percentage of sodium and chloride during the study period time (28 days). We found that sodium and chloride remaining ranged from 100.2 ± 0.26% to 107.9 ± 0.75% and from 99.04 ± 0.76% to 102.11 ± 1.71%, respectively in the short-term study; and from 101.93 ± 0.9% to 111.27 ± 2.61% and from 99.05 ± 0.94% to 110.95 ± 1.63%, respectively in the long-term study. The concentration of sodium and chloride was stable and in direct relationship with container bulging and was increased by less than 11.1% from initial concentration when stored at 50 °C, 70 °C, and cyclic temperature variation over 28 days. Sodium and chloride concentrations were comparable from the respective bags (Table 2, Table 3), suggesting no detectable loss of sodium chloride in polyolefin (PLÜMAT) bags by surface adsorption. Overall, these results indicate that normal saline solution is stable and compatible over at least 28 days and up to 70 °C in 500 mL polyolefin (PLÜMAT) containers. Under these simulated prehospital thermal storage conditions, normal saline is safe for later intravenous administration. These results can justify the allowance of using normal saline fluid in polyolefin plastic bags that have been stored inside ambulances over a tested period, rather than disposing them when not stored several days in an operational vehicle, thus decreasing waste and costs.

### 4.1 Limitations

Despite the chemical stability testing of normal saline reported in this study, other attributes of stability that are susceptible to change during storage such as biological and microbiological testing were not covered. The specific study conditions simulated and evaluated here are not necessarily applicable to all civilian and austere settings. In addition, real-life storage conditions in an operational ambulance vehicle during summer season in an arid region is yet to be assessed, to further confirm our results [33].

## 5 Conclusions

Leaching of plastic components with any of the surfactant contained in normal saline solution was not observed. Normal saline solution in polyolefin (PLÜMAT) bags that were exposed to different incubation conditions had no change in appearance (clear, no color change, and no particles) and no significant loss of sodium and chloride. In addition, polyolefin plastic bag was found to be both safe and compatible with normal saline intravenous solution under the studied conditions. There is no evidence to suggest that normal saline solution in a polyolefin (PLÜMAT) bag stored at 22 °C has any different physical and chemical properties than those exposed up to 28 days to 50 °C, 70 °C, and cyclic temperatures of 70 °C for 8 h and 22 °C for 16 h. Normal saline fluid bags made of polyolefin plastic and stored inside ambulances over a tested period are probably safe to use for patient care, but that further research through real rather than simulated environmental exposure should be conducted.

## 6 Article summary

1) Why is this topic important?

Normal saline is routinely stored and commonly used in ambulance vehicles, which may be exposed to extreme heat conditions especially in an arid climate. Data on the stability of normal saline in such stress conditions are lacking.

2) What does this study attempt to show?

This contribution provides an examination of the effect of storage under stress conditions on the stability of normal saline in a simulated prehospital emergency medical setting. In addition, the compatibility of normal saline fluid with its packaging was assessed.

3) What are the key findings?

For up to four weeks, sodium and chloride levels of normal saline were stable and compatible with polyolefin packages stored under simulated constant and cyclic extreme high temperatures.

4) How is patient care impacted?

These data can be potentially used to form updated guidelines towards the optimum storage conditions of normal saline and other intravenous fluids, towards improving the operations and logistics in prehospital emergency medical settings in wilderness and arid climates.

## Author contributions statement

OR, AM, LA, and GA conceived and designed the experiments. OR, MA, and AM performed the experiments. OR, MA, AMA, AM, and GA analyzed and interpreted the data. OR, MA, AM, LA, and GA contributed reagents, materials, analysis tools or data. OR, MA, AMA, AM, LA, and GA wrote the paper.

## Declaration of competing interest

The authors declare that they have no known competing financial interests or personal relationships that could have appeared to influence the work reported in this paper.
